# Cognitive dysfunction and its associated factors in patients with epilepsy at referral hospitals in the Amhara region: an institutional-based cross-sectional study

**DOI:** 10.3389/fneur.2025.1491716

**Published:** 2025-03-12

**Authors:** Lemlemu Maru, Yibeltal Yismaw Gela, Mihret Getnet, Dagnew Getnet Adugna, Desalegn Anmut Bitew, Ephrem Tesfaye, Hailu Aragie, Awgichew Behaile Teklemariam, Wondwosen Debebe, Mitku Mammo Taderegew, Nega Dagnew Baye, Mihret Melese

**Affiliations:** ^1^Department of Human Physiology, School of Medicine, College of Medicine and Health Science, University of Gondar, Gondar, Ethiopia; ^2^Department of Epidemiology and Biostatistics, Institute of Public Health, College of Medicine and Health Science, University of Gondar, Gondar, Ethiopia; ^3^Department of Anatomy, School of Medicine, College of Medicine and Health Science, University of Gondar, Gondar, Ethiopia; ^4^Department of Reproductive Health, Institute of Public Health, College of Medicine and Health Science, University of Gondar, Gondar, Ethiopia; ^5^Department of Biomedical Sciences, Madda Walabu University Goba Referral Hospital, Bale-Robe, Ethiopia; ^6^Department of Biochemistry, College of Medicine and Health Sciences, Debretabor University, Debre Tabor, Ethiopia; ^7^Department of Biomedical Science, Wollo University, Dessie, Ethiopia; ^8^Department of Biomedical Sciences, College of Medicine and Health Sciences, Wolkite University, Wolkite, Ethiopia

**Keywords:** cognitive dysfunction, associated factors, epilepsy, Ethiopia, MoCA

## Abstract

**Background:**

Epilepsy is a multifactorial disease characterized by spontaneous, recurrent seizures and a growing incidence of comorbid conditions such as anxiety, depression, cognitive dysfunction, and sudden unexpected death. Patients with epilepsy often experience cognitive impairment or dysfunction that can negatively affect their quality of life. There is limited research on cognitive dysfunction assessed through the Montreal Cognitive Assessment (MoCA) in the Amhara region, although the MoCA is considered superior to the Mini-Mental State Examination (MMSE). Therefore, this study aimed to assess cognitive dysfunction and identify factors associated with it in patients with epilepsy who were receiving follow-up care at referral hospitals in the Amhara region.

**Materials and methods:**

A multicenter, institutional-based cross-sectional study was conducted among patients with epilepsy who were receiving follow-up care at randomly selected referral hospitals in the Amhara region from January 2024 to July 2024. A total of 355 participants were recruited for the study using a systematic random sampling technique, achieving a response rate of 98%. Cognitive dysfunction was measured using the MoCA. Data were entered with EpiData version 4.7 and then exported into SPSS version 26 for analysis. Multivariable logistic regression analysis was conducted, and a *p*-value of ≤0.05 was considered statistically significant. The results are presented in text and tables.

**Results:**

The majority of the participants were women (52.1%). The mean age of the study participants was 31 (± 5.4) years. The prevalence of cognitive dysfunction was 29% (95% CI: 25.8, 34.5). Multivariable logistic regression analysis revealed that several factors were statistically significantly associated with cognitive dysfunction. Factors associated with cognitive dysfunction included being a rural resident (adjusted odds ratios (AOR) = 1.21; 95% CI: 1.29, 1.43), having a medical illness (AOR = 2.5; 95% CI: 2.1, 9.1), experiencing generalized seizures (AOR = 1.3; 95% CI: 1.08, 3.1), having a seizure frequency of daily to every other day (AOR = 2; 95% CI: 1.5, 9.2), experiencing seizures for more than 30 years (AOR = 1.5; 95% CI: 1.7, 7.6), and using a combination of anti-seizure drugs (AOR = 2.5; 95% CI: 1.2, 6.2).

**Conclusions and recommendations:**

In this study, a significant proportion of patients with epilepsy receiving follow-up care experienced cognitive dysfunction. Neuropsychological assessment should be emphasized in patients with epilepsy at diagnosis and early follow-up phases of the condition.

## Introduction

Epilepsy is a multifactorial disease characterized by spontaneous, recurrent seizures and a growing incidence of comorbid conditions such as anxiety, depression, cognitive dysfunction, and sudden unexpected death (1–3). It has a devastating effect on the social, cognitive, psychological, and physical aspects of life, ultimately affecting the quality of life of the patients (4, 5). Epilepsy is the most common serious chronic neurological condition, affecting 7.6 individuals per 1,000 globally across all ages, sexes, ethnicities, and social statuses, regardless of the geographic location (6). Notably, its incidence and prevalence are slightly higher in men than in women and are also higher in developing countries (7, 8).

A study conducted in Australia showed that patients experiencing epileptic seizures performed more poorly in executive function, language, and memory areas compared to those with non-epileptic seizures. Several risk factors are likely involved in the development and progression of cognitive dysfunction in patients with epilepsy (9, 10). Generalized tonic–clonic status epilepticus is one of the risk factors for cognitive dysfunction (9, 11). Other factors include the underlying etiology, recurrent seizures, structural damage that leads to secondary epilepsy, genetic variants, and molecular changes (5, 12). A study showed that anti-seizure drugs were not independently associated with cognitive function (11). Physiological causes of cognitive dysfunction associated with epilepsy include disruptions in GABAergic transmission and/or function, and abnormal presence of excitatory loops (5, 13). As a consequence of these deficits, the temporal organization of neuronal firing within networks and across structures is altered, leading to a progressive decline in cognitive function (1, 14). Therefore, restoring these circuits may help prevent seizures and cognitive deficits (3, 15, 16). The impact of epilepsy on cognition has both transient and prolonged effects, such as functional and structural abnormalities. Comorbidity with cognitive dysfunction worsens the situation (2, 3, 17).

The prevalence and incidence of epilepsy in Ethiopia are high, affecting 29.5 per 1,000 population, with an annual incidence of 64 per 100,000 inhabitants (18, 19). In countries like Ethiopia, where many individuals have limited awareness of health concerns, a significant proportion of the population seeks medical help from traditional healers for their ailments, and traditional medicine is commonly utilized. The cultural acceptance of these healers, the affordability of traditional remedies, and the challenges of accessing modern healthcare services contribute to the reliance on traditional medicine by up to 80% of individuals in developing countries (20). The occurrence of uncontrolled seizures was observed to be higher than the anticipated frequency, which ideally should be zero after 1 year of treatment. Factors related to clinical conditions and treatment have been identified as contributing to the occurrence of uncontrolled seizures (21).

Patients with epilepsy experience significant cognitive impairment that is influenced by various epilepsy-related characteristics (22). Comorbid cognitive impairment can negatively impact the quality of life of individuals with epilepsy (23, 24). However, this impairment often does not receive adequate attention and are frequently left untreated. Studies conducted in Zambia, East Gojam hospitals, and the University of Gondar Specialized Hospital have shown that nearly half of the study participants with epilepsy experienced impaired quality of life (6, 9, 25, 26). A notable incidence of cognitive impairment or dysfunction has been observed in treatment-naïve individuals with epilepsy, significantly affecting their memory, mental processing speed, and language abilities (27). Patients with epilepsy also experience sleep disturbances that contribute to cognitive dysfunction (28).

The prevalence of cognitive impairment in health institutions in Gurage Zone and South Gondar of Ethiopia was reported to be 25.6 and 69.2%, respectively (29, 30). Studies on cognitive dysfunction using the Montreal Cognitive Assessment (MoCA)—a useful tool for assessing cognition in low-to-middle-income countries, where neuropsychological tools are limited—among patients with epilepsy in the Amhara region are scarce. We aimed to assess cognitive dysfunction and identify factors associated with it in patients with epilepsy who were receiving follow-up care at referral hospitals in the Amhara region. This study may be used as a resource for clinicians emphasizing cognitive dysfunction in the treatment and follow-up of patients with epilepsy and as a baseline for researchers conducting further studies.

## Materials and methods

### Study area, study period, and study design

A multicenter, institutional-based cross-sectional study was conducted in referral hospitals in the Amhara region from January 2024 to July 2024. The Amhara region is one of the largest regions in Ethiopia. Bahir Dar is the capital of the Amhara region, which is approximately 500 km away from the capital of Ethiopia, Addis Ababa. There are eight referral hospitals in the region. This study was conducted in three randomly selected referral hospitals, each serving between 5 and 7 million people.

### Population and sample size calculation

The source population consisted of patients with epilepsy aged 18 years or older who were undergoing follow-up at referral hospitals in the Amhara region. The study population included those patients with epilepsy, aged 18 years or older, who were undergoing follow-up and were present during the data collection period.

Using the single proportion population formula, the sample size was determined based on the estimated prevalence of cognitive dysfunction, which was 69.2%, as identified in a study conducted in South Gondar (23). Since South Gondar is located in the Amhara region, the study participants shared similar sociodemographic and behavioral characteristics, disease burden, and access to healthcare as those in our study.


$$ni=\frac{{\left(Z\alpha /2\right)}^{2}*p\left(1-p\right)}{d^{2}}=\frac{{\left(1.96\right)}^{2}*0.692\;\left(1-0.692\right)}{{\left(0.05\right)}^{2}}\;\mathrm{= 329}$$

After adding a non-response rate of 10%, the total sample size was calculated to be 362, with a 5% margin of error and a 95% confidence interval.

### Sampling technique and procedure

Of the eight referral hospitals, three were selected using the lottery method.

**Figure 1:**
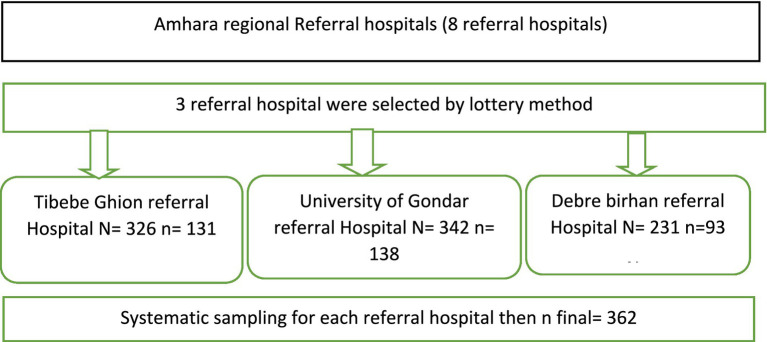
Sampling procedure for the assessment of cognitive dysfunction in epileptic patients at referral hospitals in the Amhara region, 2024.

Study participants were recruited using a systematic random sampling technique. First, a list of patients was created for each randomly selected health institution. Proportional allocation was applied to the selected referral hospitals. Finally, the study population was selected through systematic random sampling (refer to Figure 1).

### Eligibility criteria

#### Inclusion criteria

All adult epileptic patients aged 18 years and older who were undergoing follow-up at the selected referral hospitals were included in the study.

#### Exclusion criteria

Epileptic patients who were severely ill and those with observable verbal communication problems were excluded from the study.

### Study variables

The dependent variable was cognitive impairment measured using the MoCA as a dichotomous variable (Yes/No).

Independent variables were as follows: sociodemographic factors (age, sex, residence, marital status, occupation, income, and educational status); behavioral and clinical variables (substance use and medical illness); seizure-related factors (seizure frequency, duration of seizures, and type of seizures); and medication-related factors (anti-seizure drugs, frequency of medication, and comfort with medications). Comfort with medications was assessed by directly asking the study participants about their experience and elaborating on the side effects of the drugs. If the patient reported no side effects, they were considered to be comfortable with the medication, and if the patient experienced side effects, they were considered to be uncomfortable.

### Data collection tools

Data were collected through in-person interviews using an Amharic adaptation of a standardized questionnaire. Cognitive dysfunction was assessed using the MoCA, which is a sensitive and rapid assessment tool for screening patients with cognitive dysfunction. The cognitive domains assessed included visuospatial/executive, naming, attention, language, abstraction, orientation, and delayed recall. The total score was 30 points, with a score of 26 or above indicating normal cognitive function. An additional point was added for the participants with less than 12 years of education.

Cognitive dysfunction: A patient was considered to have cognitive dysfunction when their MoCA score was less than 26 (31). One point was added for the participants with less than 12 years of education.

Medical illness: To assess the history of medical illness, the respondents were asked, “Did you have any medical illness (e.g., DM, HTN, HIV/AIDS, etc.)?” The responses were recorded as yes/no.

Substance use: This included consumption of cigarettes, alcohol, or khat (9).

### Data quality control

A total of 12 BSc-level psychiatry professionals, 4 from each of the three participating hospitals, were selected and appointed as data collectors. Training was provided to both data collectors and supervisors on proper data collection techniques. The questionnaire was translated into Amharic and translated back into English to ensure consistency. Two weeks prior to data collection, a pretest was conducted on 5% (15) of the samples from Bahirdar Felege Hiwot Referral Hospital to assess the clarity of instruments. The data from the pretest were excluded from the main analysis. Based on the pretest findings, the questionnaire—particularly the structured portion—was revised and adapted. The participants gave informed consent after receiving the study information. Regular supervision was provided during the data collection, and daily checks were conducted to ensure data completeness and consistency.

### Data entry, processing, and analysis

The data were initially entered into EpiData version 4.7 and then exported to SPSS version 26 for statistical analysis. Descriptive results were presented using frequency tables and text. Bivariate analysis was performed to explore the association between the predictor and dependent variables, and a *p*-value of less than or equal to 0.25 was used as the cutoff for multivariable logistic regression. To control for confounding factors and identify the most significant variables, multivariable logistic regression analysis was conducted. The strength of the association was measured using an adjusted odds ratio (AOR) with a 95% confidence interval, and a *p*-value of less than 0.05 was considered statistically significant. The model fit was assessed using the Hosmer–Lemeshow test (*p* = 0.32). Multicollinearity among the independent variables was assessed using the variance inflation factor (VIF) and tolerance. VIF values were less than 10, while tolerance values were greater than 0.1, indicating no dependence among the independent variables.

### Ethical clearance

Ethical clearance was obtained from the Institutional Review Committee of the University of Gondar, and written permission was secured from each participating hospital. All participants provided written informed consent, and confidentiality was maintained by removing personal identifiers. The study’s purpose, benefits, and voluntary nature were explained, emphasizing the participant’s right to withdraw at any time. While no direct benefits were offered, the participants identified with cognitive dysfunction through the MoCA were referred to specialists or psychiatrists for timely and appropriate interventions, such as non-pharmacological treatments such as cognitive rehabilitation, cognitive training, and memory aids.

## Results

### Sociodemographic characteristics of the study participants

In this study, a total of 355 participants were included, achieving a response rate of 98%. The majority of the participants were women (53.8%). The mean age of the study participants was 31 years (±5.4). Approximately 44.2% of the participants were single, and 30.4% of the study participants had no formal education. The majority (58.9%) of the participants resided in rural areas, and approximately 52.4% were farmers. A total of 38.6% of the participants reported a monthly income ranging from 1,000 to 2000 ETB (see Table 1).

**Table 1:** Sociodemographic characteristics of the patients with epilepsy who were undergoing follow-up at the randomly selected referral hospitals in the Amhara region, 2024.

Study variables	Category	Frequency	Percentage
Sex	Male subjects	164	46.2
	Female subjects	191	53.8
Age	18–25	80	22.5
	26–35	115	32.4
	36–45	141	39.7
	>45	19	5.4
Marital status	Single	157	44.2
	Married	156	43.9
	Divorced	32	9
	Widowed	9	2.5
Residence	Urban	146	41.1
	Rural	209	58.9
Level of education	No formal education	108	30.4
	Primary education (1-8)	110	31
	Secondary education (9-12)	100	28.1
	Diploma and above	37	10.4
Occupation	Government employee	119	33.5
	Farmer	186	52.4
	NGO	10	2.8
	Others/unemployed	40	11.3
Income	≤1,000	94	26.5
	1,001–2000	137	38.6
	20,001–3,500	74	20.8
	>3,500	50	14

### Behavioral and clinical characteristics of the study participants

In the present study, 49% of the study participants had a history of substance use. A total of 50.1% were diagnosed with generalized tonic–clonic seizures, and 21.1% had a history of medical illness. Approximately 27% of the study participants experienced seizures daily or every other day. Of the participants, 35% used phenobarbitone. The majority of the study participants (51.3%) took their medication twice daily, and 51.3% were comfortable with their medication. The overall prevalence of cognitive dysfunction based on the MoCA was 29% (See Table 2).

**Table 2:** Behavioral and clinical characteristics of the patients with epilepsy at the randomly selected referral hospitals in the Amhara region, 2024.

Study variables	Category	Frequency	Percentage
Substance use	Yes	174	49
	No	181	51
Medical illness	Yes	75	21.1
	No	280	78.9
Type of seizure	Generalized seizures	178	50.1
	Focal seizures	177	49.9
Frequency of seizure	Daily to every other day	95	26.8
	Weekly to every other week	99	27.9
	Once in 3–4 weeks	95	26.8
	Once in the past 1–6 months	34	9.6
	6–11 months ago	23	6.5
	1–4 years ago	19	5.4
Duration of seizure (years)	≤10	118	33.2
	11–20	141	39.7
	21–30	66	18.6
	>30 (36)	31	8.7
Anti-seizure drugs	Phenytoin	124	35
	Phenobarbitone	113	31.8
	Valproate	48	13.5
	Combination	70	19.7
Frequency of medication	Once daily	173	48.7
	Twice daily	182	51.3
Comfortable with medication	Yes	173	48.7
	No	182	51.3
Cognitive dysfunction	Yes	103	29
	No	252	71

### Prevalence of cognitive dysfunction (CD)

In this study, 29% of the study participants (103 out of 355) had cognitive dysfunction (95% CI; 25.8, 34.5), with a MoCA score below 26. The mean MoCA score for patients with epilepsy who were undergoing follow-up was 27.74 (SD ± 1.76), with minimum and maximum values of 23 and 30, respectively. See Table 3 for details of the measurements of cognitive dysfunction.

**Table 3:** Values of the measurements of cognitive dysfunction based on the MoCA.

Components of the MoCA	Maximum normal value	Minimum	Maximum	Mean	SD
Visuospatial/executive	5	3	5	4.87	±0.34
Naming	3	2	3	2.98	±0.1
Attention	6	3	6	5	±0.73
Language	3	2	3	2.96	±0.17
Orientation	6	3	6	5.1	±0.71
Abstraction	2	1	2	1.98	±0.1
Delayed recall	5	3	5	4.71	±0.49
Total	30				

### Factors associated with cognitive dysfunction in patients with epilepsy

Bivariable and multivariable logistic regression analyses of cognitive dysfunction were conducted, as detailed in Table 3. In the bivariable analysis, age, residence, educational status, substance use, medical illness, type of seizure, frequency of seizure, duration of seizure, anti-seizure drugs, and frequency of medication were considered candidate variables for the multivariable analysis (*p* ≤ 0.25). The multivariable logistic regression analysis revealed statistically significant factors associated with cognitive dysfunction, such as residence (AOR = 1.21; 95% CI: 1.29, 4.43), medical illness (AOR = 2.5; 95% CI: 2.1, 9.1), generalized seizure (AOR = 1.3; 95% CI: 1.08, 3.1), seizures occurring daily to every other day (AOR = 2; 95% CI:1.5, 9.2), seizure duration greater than 30 years (AOR = 1.5; 95% CI: 1.7, 7.6), and a combination of anti-seizure drugs (AOR = 2.5; 95% CI: 1.2, 6.2) (Table 4).

**Table 4:** Factors associated with cognitive dysfunction in patients with epilepsy in the randomly selected referral hospitals in the Amhara region, 2024.

Variables	Category	CD		COR (95% CI)	AOR (95% CI)	*p*-value
		Yes *N* (%)	No *N* (%)			
Sex	Male subjects	54 (15.2)	110 (31)	1		
	Female subjects	49 (13.8)	142 (40)	1.4 (0.75, 1.68)		
Age	18–25	22 (6.2)	58 (16.3)	1	1	1
	26–35	24 (6.7)	91 (25.6)	2.4 (2.9, 20.1)	1.13 (0.8, 22)	0.15
	36–45	51 (14.3)	90 (25.3)	3.1 (2.6, 13.7)	1.5 (0.1, 9.5)	0.08
	>45	6 (1.3)	13 (3.6)	4.4 (1.8, 15)	3.2 (0.98, 12)	0.06
Marital status	Single	45 (12.7)	112 (31.5)	1		
	Married	43 (12.1)	114 (32.1)	1.2 (0.7, 5)		
	Divorced	12 (3.4)	20 (5.6)	1.4 (0.811)		
	Widowed	3 (0.8)	6 (1.3)	1.6 (0.413)		
Residence	Urban	47 (13.2)	99 (27.8)	1	1	1
	Rural	56 (15.8)	153 (43)	1.29 (1.53, 1.79)	1.21 (1.29, 1.43)*	**0.003**
Level of education	No formal education	35 (9.8)	73 (20.5)	1.5 (0.6, 12.4)	1.7 (0.2, 1.03)	0.064
	Elementary school	26 (7.3)	84 (23.7)	1.4 (0.4, 9.7)	1.6 (0.3, 1.67)	0.32
	Secondary school	32 (9)	68 (19.1)	1.3 (0.5, 4.9)	1.4 (0.1, 1.2)	0.41
	Diploma and above	10 (2.8)	27 (7.6)	1	1	1
Occupation	Government employee	37 (10.4)	82 (23)	1		
	Farmer	52 (14.6)	134 (37.7)	1.5 (0.2, 1.8)		
	NGO	2 (0.5)	8 (2.2)	1.3 (0.5, 3.4)		
	Others/unemployed	12 (3.3)	28 (7.9)	1.6 (0.9, 7.2)		
Income	≤ 1,000	32 (9)	62 (17.4)	2 (0.6, 5.3)		
	1,001–2000	43 (12.1)	94 (26.4)	1.5 (0.4, 3)		
	2001–3,500	23 (6.5)	51 (14.3)	1.4 (0. 23, 9)		
	>3,500	5 (1.4)	45 (12.7)	1		
Substance use	Yes	58 (16.3)	116 (32.7)	1.51 (1.1, 4.2)	1.6 (0.9, 5.6)	0.062
	No	45 (12.7)	136 (38.3)	1	1	1
Medical illness	Yes	37 (10.4)	38 (10.7)	3.1 (2.3, 10.7)	2.5 (2.1, 9.1)	**0.002**
	No	66 (18.6)	214 (60.3)	1	1	1
Type of seizure	Generalized	60 (16.9)	118 (33.2)	1.62 (1.1, 2.58)	1.3 (1.08, 3.1)*	**0.01**
	Focal	42 (11.8)	134 (37.7)	1	1	1
Frequency of seizure	Daily to every other day	37 (10.4)	58 (16.3)	1.81 (1.3, 12)	2 (1.5, 9.2)*	**0.047**
	Weekly/every other week	27 (7.6)	72 (20.3)	2.1 (1.8, 9.7)	1.9 (1.2, 11)	0.06
	Once in 3–4 weeks	19 (5.4)	76 (21.4)	2.3 (1.6. 9.1)	1.2 (1.62, 10)	0.19
	Once in the past 1–6 months	10 (2.8)	24 (6.8)	1.2 (1.5. 8.5)	1.1 (1.3, 9.4)	0.23
	6–11 months ago	6 (1.7)	17 (4.8)	1.9 (1.1, 13)	1.6 (1.09, 14)	0.15
	1–4 years ago	4 (1.1)	15 (4.2)	1	1	1
Duration of seizure (years)	≤ 10	37 (10.4)	81 (22.8)	1	1	1
	11–20	48 (13.5)	93 (26.2)	2.4 (1.1, 4.6)	1.8 (0.5, 5.4)	0.21
	21–30	10 (2.8)	56 (15.8)	1.53 (0.9, 12.1)	1.46 (0.3, 13)	0.25
	>30	9 (2.5)	22 (6.2)	1.6 (2.1, 5.3)	1.5 (1.7, 7.6)*	**0.04**
Anti-seizure drugs	Phenytoin	43 (12.1)	81 (22.8)	1	1	1
	Phenobarbitone	35 (9.9)	78 (21.9)	1.9 (1.64, 2.7)	1.7 (0.52, 6.6)	0.06
	Valproate	11 (3.10)	37 (10.4)	1.4 (1.84, 3.45)	1.2 (0.9, 5.4)	0.07
	Combination	14 (3.9)	56 (15.8)	3.1 (1.3, 5.6)	2.5 (1.2, 6.2)**	**0.001**
Frequency of drugs	Once daily	61 (17.2)	112 (31.5)	1	1	1
	Twice daily	42 (11.8)	140 (39.4)	1.8 (1.07, 7.8)	1.4 (0.8, 8.2)	0.052
Comfortable with drug	Yes	54 (15.2)	118 (33.3)	1	1	1
	No	49 (13.8)	132 (37.1)	1.23 (1.08, 9.5)	1.3 (0.9, 8.4)	0.14

1, reference category, Hosmer–Lemeshow value = 0.32; *, *p* ≤ 0.05; **, *p* ≤ 0.001. Bold values indicate associated factors to the dependent variable.

## Discussion

The present study identified the prevalence of cognitive dysfunction and its associated factors in patients with epilepsy. The observed prevalence of cognitive dysfunction in patients with epilepsy undergoing follow-up at the three randomly selected referral hospitals in the Amhara region was determined to be 29% (95% CI; 25.8, 34.5). This rate is comparable to studies conducted in the Gurage zone at 25.6% (29) and in Burkina Faso at 25.5% (32). This slight variation may be due to differences in the measurement of cognitive dysfunction, as these studies used the Mini-Mental State Examination (MMSE), which measures mild cognitive dysfunction. This implies that, despite a normal MMSE score, the MoCA could be used to detect cognitive dysfunction (31). On the other hand, the prevalence of cognitive dysfunction in our study was lower than that reported in a study conducted in South Gondar health institutions, where the prevalence was 69.2% (33). This variation could be due to differences in the study area, as the previous study focused on patients with epilepsy who had follow-ups in primary hospitals, which may not have sufficient resources (such as physicians and laboratory facilities) to diagnose epilepsy, compared to referral hospitals. The prevalence of cognitive dysfunction in our study was also lower than that in a study conducted in the United States, which was 60% (34). This variation may be due to differences in the study area, study design, sample size, and measurement of cognitive dysfunction.

Rural residents were twice as likely to have cognitive dysfunction compared to urban residents. This finding is supported by studies conducted in Ethiopia (33) and Burkina Faso (32). A possible reason may be that rural residents have limited access to early diagnosis, treatment, or follow-up and they may prefer to visit holy water sites or mosques, which may result in diagnostic delays and complicate the nature of the seizures (35). All of these factors may lead to cognitive dysfunction.

The patients with epilepsy who had comorbid medical illnesses were 2.5 times as likely to experience cognitive dysfunction compared to those who did not have comorbid medical illnesses. This result is in line with findings from studies conducted in the United States (34), Spain (36), Slovakia (37), and South Korea (38). This may be explained by medical illnesses such as hypertension and DM, which can cause memory loss and poor executive function (38).

The type of seizure (generalized seizures) was associated with cognitive dysfunction. The patients with epilepsy who had generalized seizures were 1.6 times more likely to have cognitive dysfunction compared to those who had focal seizures. This result is in line with findings from studies conducted in Ethiopia (29), China (39), the United States (35), Australia (17) Slovakia (37), Spain (36), and Norway (12). This may be explained by the fact that alterations in neuronal dynamics are more common in generalized seizures than in focal seizures, resulting in reduced abilities in various executive functions and the acquisition of knowledge (3, 36). The relationship between cognitive impairment and seizure characteristics is frequently perceived as one in which changes in seizure characteristics lead to changes in cognitive ability (40).

The frequency of seizures and the duration of seizures were also associated with cognitive dysfunction. This finding is supported by studies conducted in Slovenia (7), Australia (11), The Netherlands (41), the United States (35), and Egypt (22). This may be explained by alterations in the glutamate–glutamine cycle components, such as neurotransmitters and metabolites, enzymes, amino acid transporters, and glutamate receptors (1). Changes in neural networks may increase the risk of seizures and related cognitive impairment (42).

Patients taking a combination of anti-seizure drugs were 2.5 times more likely to experience cognitive dysfunction compared to those taking monotherapy. This result is in line with findings from studies conducted in Thailand (31), Australia (11), China (39), and Slovenia (7). This may be explained by the effects of a combination of anti-seizure drugs that affect memory and concentration (phenobarbitone), attention, problem-solving, and visuomotor tasks (phenytoin) (35).

## Conclusions and recommendations

The results of this study showed that a significant proportion of patients with epilepsy had cognitive dysfunction. Factors such as residence, medical illness, type of seizures, frequency of seizures, duration of seizures, and a combination of anti-seizure drugs were associated with cognitive dysfunction. Emphasis should be given to patients with epilepsy, especially to those who are rural residents, have another medical illness, experience generalized seizures, have seizures occurring daily or every other day, have had seizures for more than 30 years, and have used a combination of anti-seizure drugs. Health education, regular neuropsychological assessments, and early screening for the side effects of anti-seizure drugs should be implemented to raise awareness of cognitive dysfunction at an early stage.

### Strengths and limitations of the study

The strength of this study is that it was conducted in referral hospitals, where the diagnosis of epilepsy is more accurate due to the presence of experienced physicians, neurologists, and other laboratory setups, such as CT scans or MRI facilities. Recognizing cognitive dysfunction allows healthcare providers to develop more individualized treatment plans, ultimately improving the quality of life of the patients. This study has some limitations. First, since our tool was the MoCa, it may have been less sensitive in populations with high educational attainment. In some cases, individuals without cognitive impairment may have scored below the cutoff, leading to false positives. Second, we did not perform electroencephalograms (EEGs) or organ function tests. Finally, there may be a risk of recall bias.

## Data Availability

The raw data supporting the conclusions of this article will be made available by the authors, without undue reservation.
